# The fast azimuthal integration Python library: *pyFAI*

**DOI:** 10.1107/S1600576715004306

**Published:** 2015-03-24

**Authors:** Giannis Ashiotis, Aurore Deschildre, Zubair Nawaz, Jonathan P. Wright, Dimitrios Karkoulis, Frédéric Emmanuel Picca, Jérôme Kieffer

**Affiliations:** a European Synchrotron Radiation Facility, 71 Avenue des Martyrs, 38000 Grenoble, France; b SESAME, PO Box 7, Allan 19252, Jordan; c Synchrotron Soleil, L’Orme des Merisiers, 91190 Saint-Aubin, France

**Keywords:** powder diffraction, small-angle X-ray scattering, geometry calibration, data reduction, image analysis, GPU programming, Python, computer programs

## Abstract

*pyFAI* is an open-source software package designed to perform azimuthal integration and, correspondingly, two-dimensional regrouping on area-detector frames for small- and wide-angle X-ray scattering experiments. It is written in Python (with binary submodules for improved performance), a language widely accepted and used by the scientific community today, which enables users to easily incorporate the *pyFAI* library into their processing pipeline. This article focuses on recent work, especially the ease of calibration, its accuracy and the execution speed for integration.^1^

## Abstract 2

This article details the geometry, peak-picking, calibration and integration procedures on multi- and many-core devices implemented in the Python library for high-performance azimuthal integration.

## 1. Introduction   

Azimuthal integration is a common mathematical operation when using area detectors for recording powder diffraction and scattering patterns, which ensure larger solid angle coverage and hence a better harvest of X-­ray photons. This data reduction step is often one of the most time-consuming tasks in the processing pipeline and sometimes limits the productivity of modern synchrotron beamlines, where diffraction is used to probe samples with a point-focused beam in two-dimensional raster scans or diffraction tomography experiments using detectors capable of high frame rates.

We describe the version 0.10 of the Python library *pyFAI* (released in October 2014), which is designed for such data reduction processing, including pre-processing, image re-binning (geometry distortion, one- and two-dimensional averaging) and the auxiliary tools (Kieffer & Karkoulis, 2013 ▶). Among these tools, we will focus on the one used to calibrate the experimental setup of a powder diffraction or SAXS experiment (that comprises an area detector) by exploiting the Debye–Scherrer rings collected from a reference compound. After describing how the experimental geometry is internally represented in *pyFAI*, we present the various image analysis algorithms used to extract Debye–Scherrer rings. The peak positions are combined with the prior knowledge of a calibrant (*d* spacing) and the wavelength of the X-rays to refine of the detector’s position in space.

Once this geometry is known, azimuthal regrouping can be performed after typical corrections are done: dark-current subtraction and flat-field, solid-angle and polarization corrections are included in the standard processing pipeline. *pyFAI* implements various integration algorithms, including multiple-pixel splitting schemes, which will be described and mutually compared on the basis of speed, accuracy and memory consumption. An example will be given on how *pyFAI* can be used to decompose diffraction images into amorphous and crystalline components and how this can be applied to serial crystallography.

As *pyFAI* is a library, other projects related to *pyFAI* have been created and will be briefly described, most of them providing integrated graphical user interfaces (GUIs). Appendices contain information about the *pyFAI* project structure and an overview on how to calibrate the experimental setup parameters, as well as a description of the library and how the azimuthal integration is implemented on many-core systems using OpenCL (Stone *et al.*, 2010 ▶).

## 2. Experimental geometry   

In *pyFAI*, the basic configuration is defined by the description of an area-detector whose position in space is determined through the sample position and the incident X-ray beam.

### 2.1. Detector   

Like most other diffraction image processing packages, *pyFAI* allows the definition of two-dimensional detectors with a constant pixel size (in metres), but this approach reaches its limits with several detector types, such as multi-module and fibre optic taper coupled detectors. Large-area pixel detectors are often composed of smaller modules (*e.g.* Pilatus from Dectris, Maxipix from ESRF *etc.*). By construction, such detectors exhibit gaps between modules along with pixels of various sizes within a single module, and hence they require specific data masks. Optically coupled detectors need also to be corrected for small spatial displacements, often called geometric distortion. This is why detectors need more complex descriptions than just the pixel size. To avoid complicated and error-prone sets of parameters, detector classes have been introduced.

#### 2.1.1. Detectors classes   

Detectors classes are used to define families of detectors. In order to take the specificities of each detector into account, *pyFAI* contains about 40 detector classes. These contain a mask (invalid pixels, gaps,…) and a method to calculate the pixel positions in Cartesian coordinates. For optically coupled CCD detectors, the geometrical distortion is often described by a two-dimensional cubic spline which can be imported into the detector instance and used to calculate the actual pixel position in space.

#### 2.1.2. Nexus detectors   

Any detector object in *pyFAI* can be saved into an HDF5 file following the NeXus convention (NIAC, 2003 ▶; Könnecke *et al.*, 2015 ▶).^2^ Detector objects can subsequently be restored from disk, making complex detector definitions that are less error prone. Pixels of an area detector are saved as a four-dimensional data set: *i.e.* a two-dimensional array of vertices pointing to every corner of each pixel, generating an array of shape (*N_y_*, *N_x_*, *N*_c_, 3), where *N_x_* and *N_y_* are the dimensions of the detector, *N*_c_ is the number of corners of each pixel, usually four, and the last entry contains the coordinates of the vertex itself. This kind of definition, while relying on large description files, can address some of the most complex detector layouts:

(i) hexagonal pixels (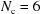, *e.g.* Pixirad detectors)

(ii) curved/bent imaging plates (*e.g.* Rigaku)

(iii) pixel detectors with tiled modules (*e.g.* some Xpad detectors from ImXpad)

(iv) semi-cylindrical pixel detectors (*e.g.* Pilatus12M from Dectris)

### 2.2. Geometry   

**Figure 1:**
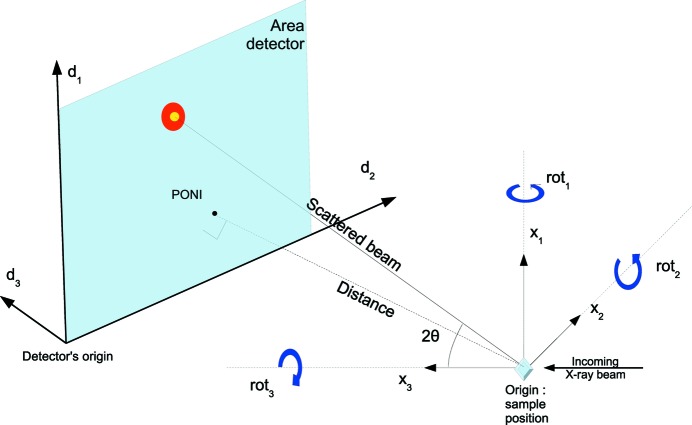
The geometry used by *pyFAI* is inspired by *SPD* (Boesecke, 2007 ▶).

In *pyFAI*, the experiment geometry is determined by the position of the detector in space, the origin being located at the sample position, more precisely, where the X-ray beam crosses the diffractometer main axis. The detector being a rigid body, its position in space is described by six parameters: three coordinates and three rotations (Fig. 1 ▶). In *pyFAI*, the beam centre is not directly used as it is ill-defined with highly tilted detectors. Like *SPD* (Boesecke, 2007 ▶), we use the orthogonal projection of the origin on the detector surface called the PONI (for point of normal incidence). For nonplanar detectors, the PONI is defined in the plane 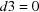 in the detector’s coordinate system. The sample-to-detector distance is defined as the origin–PONI distance (abbreviated dist), and the PONI coordinates (abbreviated poni1 and poni2) are measured in the detector’s reference system (origin at the lower left corner of the image, looking from the viewpoint of the sample). As the pixel size may not be constant, all three distances (dist, poni1 and poni2) are given in metres. The three rotations (named rot1, rot2 and rot3, in radians) correspond to the rotations along the three orthogonal axes around the origin (sample position) in this order: vertical axis, horizontal axis and finally along the beam axis.

When all rotations are zero, the detector is in transmission mode with the incident beam orthogonal to the detector’s surface. The choice of SI units may look cumbersome or odd to users familiar with other tools like *FIT2D* (Hammersley *et al.*, 1996 ▶) or *SPD* (Boesecke, 2007 ▶). To address such issues, the geometry used in *pyFAI* can be exported to and imported from parameter sets compatible with other software packages. Geometries used in other codes can be promptly included in *pyFAI* to ease comparison of results and cross-validation of approaches.

### 2.3. Binning   

One of the strengths of the above geometry is the capability of performing binning operations on the detector without having to recalibrate or recalculate the position in space. All *pyFAI* detector classes have a binning option available that can increase the pixel size and divide the detector shape accordingly. This works even for detectors that require distortion correction: *pyFAI* can bin or un-bin the spline describing the distortion, on the fly, the position of the PONI being independent of the pixel coordinates.

## 3. Calibration   

The calibration of the detector position is performed using the Debye–Scherrer rings collected from a reference powder called the calibrant. The rings are extracted (see §[Sec sec3.2.1]3.2.1) and control points are placed at the local maxima on the rings. The geometry of the experiment is obtained from a least-squares fitting of the 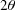 angles. In this work we will call them ‘rings’ even if, for a planar detector, they are actually the conic intersections of the X-ray beam cones with the detector plane. *pyFAI* does not assume that rings are conic sections (the detector could be nonplanar) and is able to optimize the geometric parameters of a wide range of experiments. The support for the geometry refinement of nonplanar detectors is still under development.

### 3.1. Calibrant   

*pyFAI* provides ten calibrant descriptions covering the most used ones: ceria, corundum, gold, lanthanum hexaboride and silicon for powder diffraction measurements; silver behenate, tetradecanol and *para*-bromobenzoic acid for small-angle scattering experiments. Any file containing *d*-spacing values in ångströms can be used as calibrant and loaded into the *Calibrant* class. The *calibrant* object is in charge of calculating the reference aperture of the diffraction cones (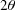), provided the wavelength or energy is known.

### 3.2. Peak picking   

With the advent of micro- and nano-focused beams at modern synchrotron facilities (Riekel *et al.*, 2010 ▶), fewer crystals get hit by the beam going through the sample, causing the Debye–Scherrer rings to be spotty. As grinding of the reference powder is not advised (it would broaden the peaks and may even introduce strain), we decided to address this issue by further analysing and reconstructing the Debye–Scherrer rings. An alternative approach would be the use of single-crystal indexing techniques, using for example the *Fable* software (Oddershede *et al.*, 2010 ▶) as demonstrated for diffraction tomography experiments (Bonnin *et al.*, 2014 ▶).

#### 3.2.1. ‘Massif’ extraction   

‘Massif’ extraction allows a clear separation between regions containing high photon counts (rings) and background. This is done by calculating the difference between the image and a blurred version of the same image, using a Gaussian blur filter (of width σ). The borders of high-intensity regions (called massif) feature negative intensities in the difference image, so positive regions are labelled as (fractions of) a ring. Peaks, *i.e.* local maxima, are sampled within the same region and belong to the same ring. The width of the Gaussian, σ, in pixel units, has to be larger than the typical distance between two peaks within a ring and smaller than the distance between two rings. *pyFAI* takes a heuristic approach to guess an acceptable parameter value in most cases, while providing also a manual override through the command line argument −gaussian=sigma.

#### 3.2.2. Subpixel accuracy   

Subpixel accuracy is often needed when measuring strains in materials, as highlighted by Borbely *et al.* (2014 ▶). The accuracy on the peak position is obtained using a second-order Taylor polynomial of the intensity in the neighbourhood of the peak position 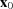: 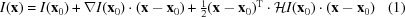
where *I*, 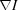 and 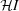 are the scalar field of intensity, its gradient (vector) and Hessian (matrix), respectively, measured at the maximum pixel position. Differentiating equation (1), one obtains
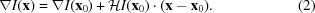
The position of the actual maximum 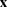 is obtained by substituting 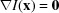 in equation (2). Hence, 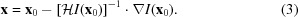
These derivatives, 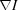 and 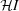, are numerically evaluated on a 3 × 3 neighbourhood (smallest possible size to calculate the Hessian matrix). With noisy data, it could happen that 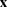 is far away from 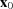 (more than one pixel), which is obviously wrong. In such cases, 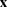 is taken as the centre of mass of the 3 × 3 neighbourhood around 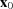 (less precise, but more robust).

#### 3.2.3. Blob detection   

Blob detection is a computer vision method which allows peak picking to be performed without *a priori* knowledge of the intensity values in the image. This feature is essential, as diffraction images exhibit a very large dynamic range.

The diffraction image is sequentially blurred using Gaussian filters, the width of which, σ, follows the geometric series 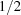, 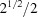, 1, 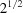, 2, 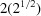,…. From each image blurred over a scale σ, the subsequent blurred image (over 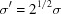) is subtracted to create a difference of Gaussians image (called DoG) which highlights the features of the image with a typical size σ. A three-dimensional scale space (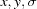) representation is created from the DoG images.

This method provides not only the locations of the peaks (as local maxima in scale space) but also the typical size of the peaks. Peak position, scale and intensity are refined as described in §[Sec sec3.2.2]3.2.2, extended to the three-dimensional scale space.

To keep the computation time reasonable, the implementation of the blob detection relies on Gaussian convolution in real space (*i.e.* without Fourier transform), separated in the horizontal and vertical directions, with small convolution kernels of width 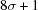. To prevent an excessive growth of the window width, a pyramid of Gaussians is built by binning blurred images by a factor 2 when reaching 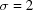.

The drawback of this algorithm, besides the computation time, is its very high sensitivity to noise in flat regions. This is why blob detection is only used in the recalibration procedure to extract all peaks in a region of interest, as determined from an approximate geometry. Moreover this algorithm cannot detect peaks the width of which is smaller than 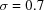 (which corresponds to three pixels).

### 3.3. Graphical user interface for calibration   

**Figure 2:**
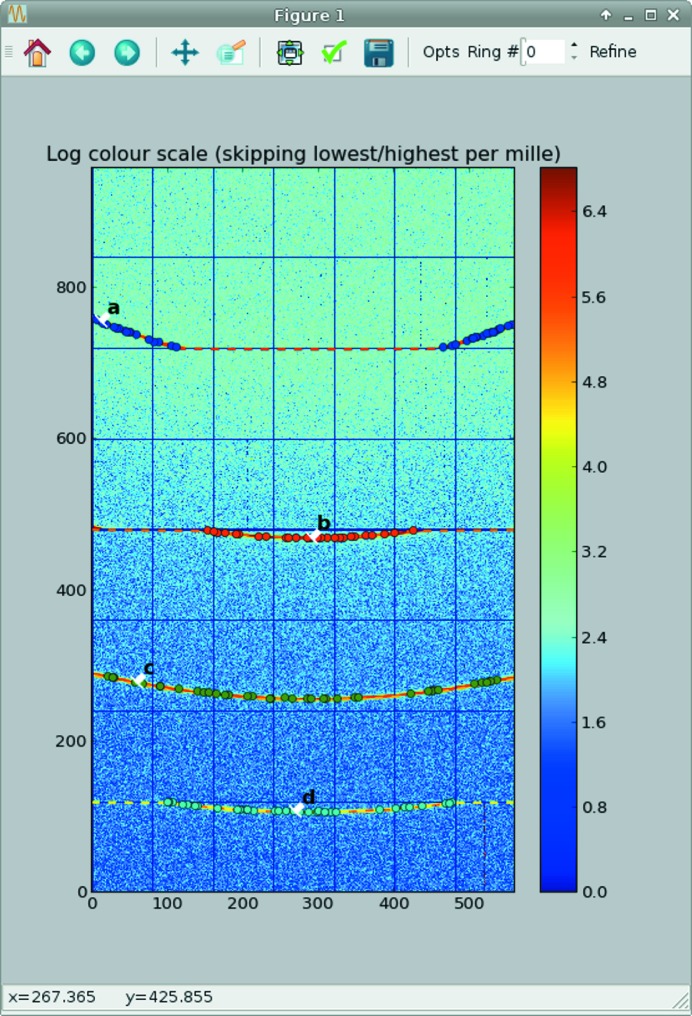
The *pyFAI* calibration window: manual peak picking and ring assignment can be performed though it. The data correspond to a lanthanum hexaboride (LaB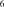) calibrant on the Cristal beamline at Synchrotron Soleil taken at 18.57 keV on an Xpad S540 flat pixel detector tilted vertically by about 15°. This detector presents large (vertical) gaps between modules, explaining the incomplete arcs of rings *a*, *b* and *d*. Extracted control points are marked with dots, one colour per group (assigned to a letter), and the fitted iso-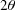 contours are overlaid as dashed lines (red, orange and yellow coloured). The iso-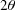 contour plot is not smooth because of gaps in the detector, explaining the incompleteness of some rings.

Only a minimalistic GUI (called *pyFAI-calib*; see Fig. 2 ▶) is provided for peak picking, with visual assignment of the ring number. A rough estimate of the geometry is usually obtained *via* a mouse click on two of the innermost rings with a ‘usual’ transmission setup. For more challenging setups (small sensitive area, tilted detectors, spotty rings,…) like the one presented in Fig. 2 ▶, more rings may be needed. The four groups of coloured dots correspond to the control points (peaks) extracted using the algorithm described in §[Sec sec3.2.1]3.2.1 (obtained from five mouse clicks). Each group of points is assigned to a diffraction ring (using the spin box in the menu bar). The refinement is performed to minimize the error in 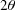 (squared) by using the sequential least-squares programming function (*scipy.optimize.min_slsqp*) from *SciPy* (Jones *et al.*, 2001 ▶). After the refinement of the geometry, the iso-contour of the refined 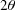 array is superimposed on the diffraction image. These are the four dashed lines drawn on Fig. 2 ▶ to mark where Debye–Scherrer rings are expected, allowing a visual validation of the calibration.

From this initial rough calibration, *pyFAI* enables the user to perform many operations in the command line interface mode, like setting, constraining, fixing and refining parameters, extracting a new set of key points, or performing the integration. The complete set of options is described in Appendix *B* [App appb].

## 4. Azimuthal integration   

The core task of *pyFAI*, as the name suggests, is to perform one- or two-dimensional azimuthal (and radial) integration as fast as possible. To achieve good performances in a Python environment, several binary extensions are used to enable multi-threading or even many-core acceleration [*i.e.* graphics processors units (GPUs) and Intel Xeon Phi accelerators]. More details on the techniques used to speed up the code, especially on the GPU porting, are described by Kieffer & Ashiotis (2014 ▶) and briefly summarized in Appendix *D* [App appd].

### 4.1. Programming interface for azimuthal integration   

The initial idea behind *pyFAI* was to provide an easy way to perform azimuthal/radial integration for scientists, ideally in a single command. In the following code snippet we show how this is done:
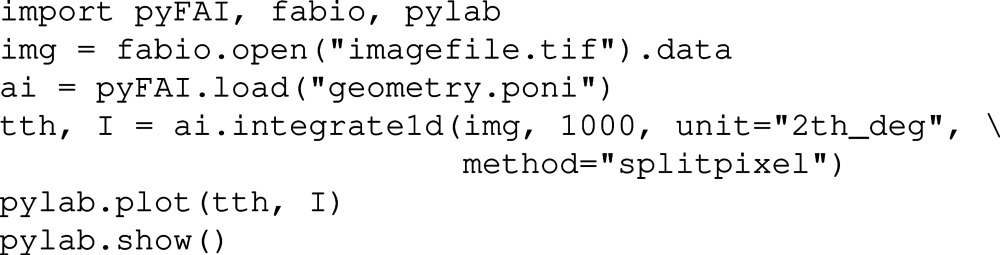


In the first line, three key libraries are loaded: *Fabio* (Knudsen *et al.*, 2013 ▶) to read images, *Matplotlib* (Hunter, 2007 ▶) to display the results and *pyFAI* itself to be able to perform azimuthal integration. In the second and third lines, the image and the geometry are loaded. The two last lines are meant to display the result.

In this snippet, the most crucial part is the fourth line, in which the image *img* is azimuthally integrated over 1000 bins with conversion into the output space, which is the cone aperture (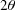) given in degrees. Other output units like the scattering vector magnitude *q* or the radius *r* (in the detector plane) are available. By the *method* keyword one can select the algorithm to be used.

### 4.2. Pixel-splitting schemes and implementation   

*pyFAI* implements a dozen azimuthal integration procedures which can be classified according to the way the integration is performed and which pixel-splitting scheme is used (see Table 1 ▶).

#### 4.2.1. Histogram *versus* lookup table   

The naive way to integrate data (also called ‘direct integration’) is to treat an image pixel by pixel, very much like a histogram. This is a scatter operation, which is hard to parallelize but cheap as to memory occupation. Using a scatter to gather transformation, the azimuthal integration for a given geometry can be stored into a lookup table (LUT) and applied like a sparse-matrix-times-dense-vector multiplication (sometimes called ‘backwards integration’). Whilst being much more memory consuming, this implementation is effective in terms of parallelization and speed. The compressed row storage (CSR) matrix representation is now used instead of the LUT and generates a smaller memory footprint.

#### 4.2.2. Three pixel-splitting schemes   

Three pixel-splitting schemes are available in *pyFAI* and define the way photons counted by a pixel are assigned to the various histogram bins, especially when the pixels are large (like on Pilatus detectors):

(1) No splitting: the full intensity is assigned into a single bin (Dirac like shape), the one at the middle of the pixel (like in the histogram).

(2) Bounding box splitting: the pixel is abstracted by a simpler rectangular box oriented parallel to the radial and azimuthal directions.

(3) Tight/full pixel splitting: the only assumption made is that pixel edges are deemed to be straight lines. This is also known as polygon-based interpolation (van der Walt, 2010 ▶).

**Figure 3:**
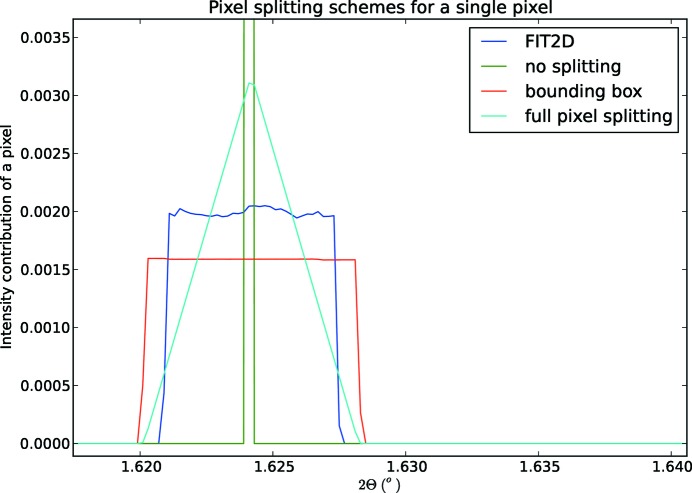
Contribution to a powder diffraction pattern from a single pixel, showcasing the different pixel-splitting algorithms. *pyFAI* implementations are compared with the corresponding *FIT2D* algorithm.

Fig. 3 ▶ displays the way a single pixel is split into a large number of bins using the three schemes explained above. The way *FIT2D* splits pixels has been added for comparison: it looks similar to the bounding box pixel splitting but there are differences in the implementation details.

#### 4.2.3. Speed and memory consumption   

Table 1 ▶ lists the various available implementations together with their execution speed and the memory footprint for integrating a 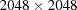 pixel image into 1000 bins.

#### 4.2.4. About error propagation   

During the regrouping process, *pyFAI* can propagate errors, assuming that the initial pixel-wise variance is known, for example as extracted from a multi-frame experiment. Besides this, two single-frame variance estimators are available. The Poisson model assumes that the variance within a pixel is equal to the raw signal and propagates it. The second estimator postulates the isotropic distribution of the signal and calculates the variance within all pixels contributing to a single output bin.

While pixel splitting provides smoother results, any pixel-splitting scheme introduces some serial correlation between neighbouring bins, resulting in an overestimation of the propagated error, as described by Yang *et al.* (2014 ▶). Nevertheless, this effect is often negligible owing to the point-spread function of typical area detectors.

### 4.3. Graphical user interface for azimuthal integration   

**Figure 4:**
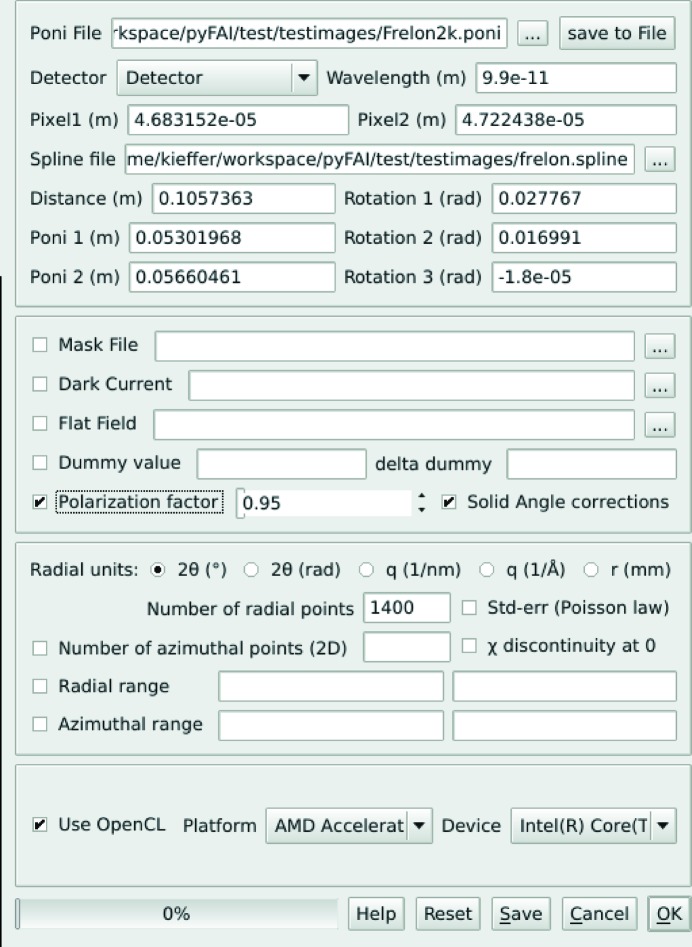
Graphical interface for performing azimuthal integration on a set of images.

A minimalistic GUI, called *pyFAI-integrate*, is shown in Fig. 4 ▶. It illustrates most of the features available in *pyFAI*. The top frame displays the geometric description of the experiment. The middle frame targets the per-pixel corrections to be applied: dark current subtraction, flat-field correction, polarization and solid angle effects, and static and dynamic masking. The check boxes next to each field are used to toggle the given correction. The third frame displays information about the output format, and the number of bins in the radial and azimuthal directions, together with the selection of the integration output space (these are mandatory). The bottom frame allows an OpenCL device (CPU/GPU) to be selected for use in the computation.

## 5. Application examples   

Azimuthal regrouping and its inverse transformation (assuming uniform intensity distribution throughout the azimuthal angles) can be performed using *pyFAI*, which offers many opportunities for applications.

### 5.1. Diffraction image generation   

Once the geometry has been defined (*i.e.* by loading a *PONI-file*), the 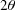 and χ positions of every single pixel of the detector are known. If one assumes signal isotropy along the azimuthal angle range (like an ideal powder without preferred orientation), two-dimensional diffraction patterns can be generated as illustrated in the example below: 
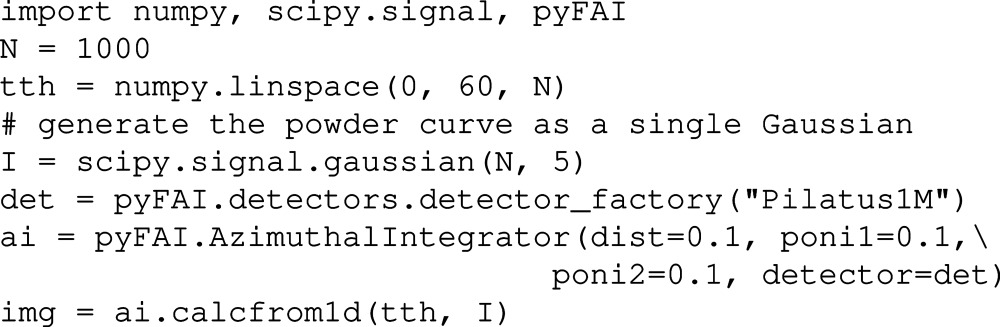


The method *calcfrom1d* is available from any *Azimuthal­Integrator* or *Geometry* class instance. It is used together with a *calibrant* object to simulate a diffraction image suitable to test *pyFAI* or other calibration codes (for example, to validate the geometry transformation from one program to another).
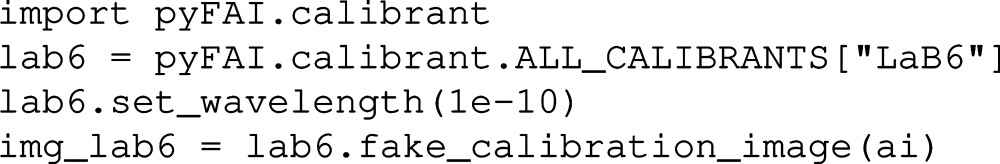


In the above code snippet, second line, a reference sample, LaB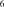, is chosen from the list of calibrants known to *pyFAI* before the wavelength is set. Once combined with the geometric information, the *calibrant* object is able to generate a two-dimensional *NumPy* (Oliphant, 2007 ▶) array containing the simulated Debye–Scherrer diffraction rings, which can be saved or displayed on the screen. The *fake_calibration_image* method takes more parameters to help set the *U*, *V* and *W* parameters from Caglioti’s formula (Caglioti *et al.*, 1958 ▶) to include the broadening of peaks according to the simple resolution function. In *pyFAI*, only the *d*-spacing values of the calibrants are stored, and thus the reconstructed image will have all rings with the same intensity (once integrated).

### 5.2. Image offset and validation of the calibration   

By regenerating a two-dimensional diffraction image from the integrated powder pattern one can assess the quality of the calibration used for the integration. The calibration tool, *pyFAI-calib*, includes a ‘validate’ command which evaluates the spatial offset between the two-dimensional diffraction image and the image regenerated from the integrated pattern, using a classical phase correlation algorithm. This determines the precision of the PONI localization, which can be better than a tenth of a pixel, when calibrating images with continuous rings (*i.e.* not spotty) and with a mask large enough to remove the beam stop and all parasitic scattering.

### 5.3. Amorphous background removal   

The *pyFAI* azimuthal integrator features a *separate* method for separating automatically a background featuring an azimuthal symmetry (amorphous scattering or powder ring) from the Bragg peaks.

Based on what was described by Kieffer & Wright (2013 ▶), two-dimensional azimuthal integration is performed on the input image. The output two-dimensional image is filtered along the azimuthal χ axis using a percentile (often the median) filter to reconstruct the powder diffraction curve without the sharp Bragg spots. The number of points in the azimuthal and radial directions as well as the percentile value can be adjusted, but the default values are in general reasonably good.

**Figure 5:**
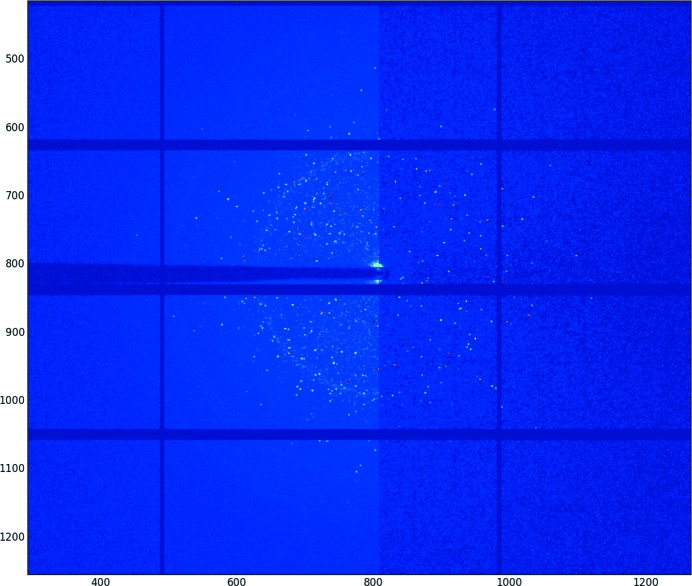
Automatic removal of the amorphous signal (ice ring) from Bragg peaks in a protein crystallography experiment (data from beamline ID23-2 at the ESRF).

The reconstructed two-dimensional image corresponds to the amorphous/powder/isotropic component of the input image and the subtraction of this image from the raw data contains only the signal coming from large crystals. Fig. 5 ▶ (left hand side) presents a close-up of protein single-crystal data recorded on a Pilatus3-2M detector (image taken at the ID23-2 beamline of the ESRF). A diffuse amorphous halo is clearly visible. After using the automatic amorphous background removal, which takes into account the mask needed for such pixel detectors, only Bragg peaks remain (right hand side of the image).

#### 5.3.1. Application to serial crystallography   

In serial crystallography experiments, tiny crystals in their solvent are moved into the X-ray beam (using a jet or moving a sample holder) and scattering data are acquired continuously, using a fast detector (from dozens of Hz to kHz). These experiments produce a huge quantity of data while only a small fraction of the frames contain some diffraction signal. *pyFAI* has been integrated into the processing software *NanoPeakCell*, which provides a graphical interface for frame selection in serial crystallography. *pyFAI* has also been integrated into the *LImA* data acquisition system (Homs *et al.*, 2012 ▶), where the quantity of single-crystal diffraction data within each frame can be assessed and a decision taken on whether to save a given frame or not. This way, a huge amount of disk space and network bandwidth can be saved.

## 6. Related work   

Currently, the *pyFAI* library runs either as a standalone application or embedded in other software on several beamlines at the ESRF to perform azimuthal/radial integration online:

(*a*) inside the *LImA* image acquisition library, running on the computer controlling the camera;

(*b*) in one dedicated data analysis server like *EDNA* (Incardona *et al.*, 2009 ▶) in the case of the BioSaxs beamline, BM29 (Pernot *et al.*, 2013 ▶), or the Dahu server at the TRUSAXS beamline, ID02.

Other institutes have independently integrated *pyFAI* into their processing pipelines: *NanoPeakCell* (developed at IBS by N. Coquelle), *PySAXS* (developed at CEA by O. Taché), *Dpdak* [developed at the Petra III synchrotron by G. Benecke *et al.* (2014 ▶)] and *Dioptas* (developed at the APS synchrotron by C. Prescher). Most of these software packages offer a GUI to facilitate the data processing for a specific type of experiment.

## 7. Conclusion and future work   

In this work, we have described the improvements in v0.10 of the *pyFAI* library, focusing on the detector representation in space, ring extraction algorithms and pixel-splitting schemes for azimuthal integration. The number of independent projects now relying on *pyFAI* proves that it fulfils a number of needs in the scientific community.

On the other hand, there are plenty of unresolved issues: all algorithms designed to perform azimuthal integration are not yet implemented in two dimensions. Is it possible to address the error propagation issue while keeping pixel splitting? Can all algorithms used in *pyFAI* be ported to GPU to offload the processor? The GUIs for calibration and integration, while helpful, are really minimalistic. The automatic ring extraction using computer vision techniques could be improved and the calibration might be fully automated. The functionality relating to the geometry refinement of nonplanar detectors is not yet complete. The version number (v0.10) clearly indicates that a great wealth of work has been done but also yields a warning about possible changes in the programming interface in future versions for encompassing numerous new features. The open-source nature of the project means that such changes will be fully visible and is intended to encourage contributions from the community.

## Figures and Tables

**Figure 6:**
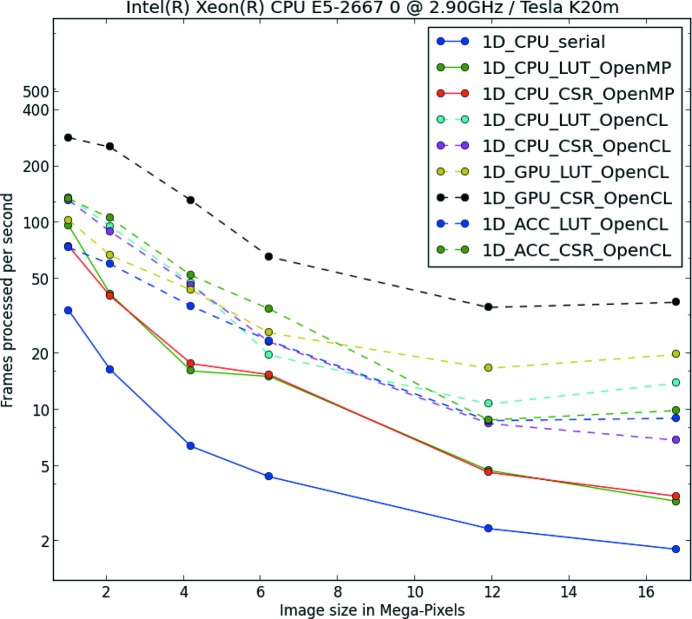
Comparison between the various *pyFAI* algorithms performing azimuthal integration.

**Table 1:** Methods available within *pyFAI* for azimuthal integration, along with their speed and memory footprint Measurements were performed on a 3GHz quad-core computer using a 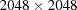 pixel image.

Pixel splitting	Direct histogram	Lookup table (reverse)
No splitting	numpy (889ms 336MB)	CSR nosplit (48ms 330MB)
	cython (361ms 323MB)	
Bounding box	splitbbox (129ms 343MB)	splitBBoxLUT (59ms 327MB)
		CSR bbox (52ms 330MB)
Tight splitting	splitpixel (516ms, 480MB)	CSR full (51ms, 502MB)

## Footnotes

2 Slightly extended as *pyFAI*’s detector definition is richer than the one provided by the NIAC.

## Appendix A Project structure

*pyFAI* is an open-source project licensed under the General Public License (GPL v3+). It is mainly written in Python (v2.6, 2.7, 3.2 or newer) and is heavily reliant on the Python scientific ‘ecosystem’: *NumPy* (Oliphant, 2007 ▶), *SciPy* (Jones *et al.*, 2001 ▶) and *Matplotlib* (Hunter, 2007 ▶). It exhibits high performance in image treatment and azimuthal/radial integration thanks to Cython (Behnel *et al.*, 2011 ▶) and PyOpenCL (Klöckner *et al.*, 2012 ▶). PyOpenCL remains an optional dependency; therefore all OpenCL code features a Python or Cython implementation as well. For the sake of consistency with other ESRF software projects (Solé *et al.*, 2007 ▶), the GUI was developed using *PyQt* (or *PySide*). The project is hosted on GitHub (https://github.com/pyFAI), which provides the issue tracker in addition to code hosting. A *pyFAI* mailing list is available: pyfai@esrf.fr (send ‘subscribe pyfai’ by e-mail to sympa@esrf.fr to subscribe to the mailing list). New features and changes in the API are discussed there. Originally developed under Linux, the software is also tested and supported on other operating systems like Windows and MacOSX. To ease the distribution, the software is available on the PyPI package repository (http://pypi.python.org) and as an official Debian package and is included in other well known Linux distributions like Ubuntu.

Everyone is welcome to fork the project and adapt it to his/her own needs: CEA Saclay, Soleil, DESY and APS have already done so. Collaboration is encouraged and new developments can be submitted and merged into the main branch *via* pull requests (on the GitHub interface).

While there are a couple of official releases every year (better tested versions), *pyFAI* features a comprehensive test suite and uses a continuous integration mechanism to ensure that any snapshot of the master branch provides valid results.

## Appendix B Calibration tool

The *pyFAI* calibration tool, called *pyFAI-calib*, has been available along with the integration tool since the very beginning of the project, because having the same geometry module for both calibration and integration has been a top priority in the project’s specifications. The geometry file (commonly called *PONI-file*) is updated at each optimization step and delivers the whole description of the experiment (together with time stamps, making it easy to edit by hand). This single-file geometry avoids copy-and-paste errors of spatial coordinates.

### b1. Calibration command line interface

While the GUI for peak picking has significantly improved (Fig. 2 ▶), the command line interface used for the optimization process has become more versatile.

Starting from an initial coarse calibration, *pyFAI* allows for performing many operations to refine all parameters: distance, centre position, rotation of the detector and, optionally, the wavelength (all units are in the SI). The following commands are available:

(*a*) help: shows the help message

(*b*) abort: quits the program directly

(*c*) assign: changes the assignment of groups to rings

(*d*) done: performs the azimuthal integration and quits

(*e*) get: prints out the value of the requested parameter,

(*f*) set: defines the value of the selected parameter,

(*g*) bound: selects the region of validity for a given parameter,

(*h*) bounds: reviews and modifies the region of validity for all parameters

(*i*) fix: prevents the parameter from being refined,

(*j*) free: allows the parameter to be refined,

(*k*) refine: re-runs the least-squares refinement

(*l*) validate: estimates the accuracy of the calibration on the whole image by overlaying and correlating the raw image with the back-projected integrated pattern

(*m*) recalib n blob: extracts a new set of control points from the *n* innermost rings; the optional parameter blob selects the ring extraction algorithm to use

(*n*) reset: sets the geometry to its default values (centred orthogonal detector)

(*o*) show: prints out the current geometry parameters on the screen

(*p*) integrate: performs the one- and two-dimensional integration and displays it in a separate window to validate the quality of the calibration

If the initial calibration is correct, like in Fig. 2 ▶, the procedure to get a perfect calibration should be ‘recalib  done  ’. If the predicted rings become too large or too small compared to the actual ones, the wavelength can be refined. For this purpose, it is advisable to extract as many rings as deemed reasonable using recalib n then run free wavelength and refine. Since distance and wavelength are heavily correlated, it is important to take into account as many rings as possible at high angle. The validate option allows measurement of the offset between the actual diffraction image and an image generated from the refined geometry using phase correlation. In most cases it is possible to attain a precision of about one-tenth of a pixel for determining the PONI position.

### b2. Automatic distance and centre calibration

The calibration procedure has been automated for the macromolecular crystallography beamlines (MX) at the ESRF. The sample-to-detector distance and the beam centre need to be input in the header of the collected images in order to process them automatically. As the area detector is on a moving stage, the distance and centre position change at every data collection. *Via* the *MX-calibrate* tool of *pyFAI*, it is possible to calibrate the geometries of a set of images recorded at various distances using the Debye–Scherrer rings of a reference compound. After subsequently performing the peak picking using blob detection (as described in §[Sec sec3.2.3]3.2.3) and the least-squares refinement of the geometry on every input frame, the program returns distances and beam centre positions as a function of the detector motor position, without manual intervention.

## Appendix C Layers in the *pyFAI* library

*pyFAI* has multiple entry points depending on the type of application and the user skills.

### c1. Top level graphical application

A couple of top level applications, *pyFAI-calib* and *pyFAI-integrate*, provide a graphical user interface to inexperienced users. These interfaces are the least flexible ones and, because they are GUIs, are not tested against regression.

### c2. Top level application

A bunch of simple scripts are provided with *pyFAI* for various kinds of processing (like *pyFAI-saxs*, *pyFAI-waxs* and *diff_tomo*), which can be integrated into shell scripts for batch processing.

### c3. Top level Python interface

*pyFAI* provides at the top level the *AzimuthalIntegrator* object, which can be created from a *PONI-file* and used to integrate data. Pre-processing options (dark, flat, mask, solid angle and polarization correction) can be passed to the *integrate1d* or *integrate2d* methods. These methods also ‘know’ how to save processed data on disk. This is probably the most flexible level of use of *pyFAI*, and the best tested one. Moreover, nothing from the underlying levels is hidden.

### c4. Mid level API

Below the *AzimuthalIntegrator* are the geometry calculation, the detector description, calibrants, geometry refinement,…. These modules are implemented in Python using *NumPy*, but often a second implementation exists in Cython for performance purposes. The equivalence of these implementations is a core target of the *pyFAI* test suite.

### c5. Regridding/histogramming engines

These number-crunching engines are typically written in Cython or OpenCL with a Python binding and can only be accessed from the Python level. They expose only the core number-crunching routines for integration or distortion correction.

The *pyFAI* project is very modular and can be accessed at various levels depending on the user’s needs.

## Appendix D Parallel implementations using OpenCL

Azimuthal integration, like many computationally intensive parts in *pyFAI*, was written as an OpenCL kernel and interfaced to Python *via* PyOpenCL (Klöckner *et al.*, 2012 ▶). PyOpenCL provides a shared execution model which is effective both on usual processors (CPUs), on graphics cards (GPUs) and on accelerators like the Intel Xeon Phi.

### d1. Azimuthal integration

The direct azimuthal integration (histogram) is basically a scatter operation which requires extensive memory locking (inefficient over many threads). To overcome this limitation, pixels have been associated with the output bin of the histogram and stored in a LUT, making the integration look like a simple (if large and sparse) matrix–vector product (Kieffer & Wright, 2013 ▶). The sparse matrix CSR format is now used in *pyFAI*, using only half of the space that the LUT previously used (Kieffer & Ashiotis, 2014 ▶). Furthermore, all threads within a workgroup collaborate to calculate the matrix–vector product *via* a so-called ‘parallel reduction’, ensuring additional speedup (especially on GPUs). The compensated algebra (Kahan summation) is kept to maintain the accuracy of the calculation while using single precision (32 bit) floating point arithmetic.

### d2. Performance

Fig. 6 ▶ shows the performance of *pyFAI* in terms of frames processed per second *versus* the input image size (in semi-logarithmic scale). The computations were run on a dual-socket Intel Xeon E5-2667 (2 hexacore @2.9Ghz) computer with an Nvidia Tesla K20 GPU and an Intel Xeon Phi accelerator.

In this benchmark, four groups of curves can be identified:

(1) The lower continuous blue curve presenting the serial Cython code using histograms (corresponding to the ‘splitbbox’ method), which is the slowest implementation (even if it is 7× faster than a *NumPy* implementation).

(2) The red and green continuous curves, which correspond to the two parallel Cython implementations for lookup-table-based integration (using LUT and CSR representation).

(3) The group of dashed curves that represent the OpenCL optimized code running on 12 CPU cores, 60 cores from the accelerator and GPU (LUT implementation).

(4) The upper dashed black curve, hence the fastest, corresponding to the CSR sparse matrix multiplication algorithm implemented in OpenCL and running on the Tesla K20 card. It is two times faster than any other implementation: a 4096 × 4096 pixel image can be processed in less the 19 ms, *i.e.* 885 megapixels per second. This gain in performance is obtained from the collaborative partial reduction of all threads within a workgroup.
